# ProteoLens: a visual analytic tool for multi-scale database-driven biological network data mining

**DOI:** 10.1186/1471-2105-9-S9-S5

**Published:** 2008-08-12

**Authors:** Tianxiao Huan, Andrey Y Sivachenko, Scott H Harrison, Jake Y Chen

**Affiliations:** 1 School of Informatics, Indiana University – Purdue University, Indianapolis, IN 46202, USA; 2 Indiana Center for Systems Biology and Personalized Medicine, Indianapolis, IN 46202, USA; 3 Department of Computer & Information Science, Purdue University, Indianapolis, IN 46202, USA; 4 College of Life Sciences, Shandong University, Jinan 250100, China; 5 the Broad Institute of MIT & Harvard University, Cambridge, MA 02141, USA

## Abstract

**Background:**

New systems biology studies require researchers to understand how interplay among myriads of biomolecular entities is orchestrated in order to achieve high-level cellular and physiological functions. Many software tools have been developed in the past decade to help researchers visually navigate large networks of biomolecular interactions with built-in template-based query capabilities. To further advance researchers' ability to interrogate global physiological states of cells through multi-scale visual network explorations, new visualization software tools still need to be developed to empower the analysis. A robust visual data analysis platform driven by database management systems to perform bi-directional data processing-to-visualizations with declarative querying capabilities is needed.

**Results:**

We developed ProteoLens as a JAVA-based visual analytic software tool for creating, annotating and exploring multi-scale biological networks. It supports direct database connectivity to either Oracle or PostgreSQL database tables/views, on which SQL statements using both Data Definition Languages (DDL) and Data Manipulation languages (DML) may be specified. The robust query languages embedded directly within the visualization software help users to bring their network data into a visualization context for annotation and exploration. ProteoLens supports graph/network represented data in standard Graph Modeling Language (GML) formats, and this enables interoperation with a wide range of other visual layout tools. The architectural design of ProteoLens enables the de-coupling of complex network data visualization tasks into two distinct phases: 1) creating network data association rules, which are mapping rules between network node IDs or edge IDs and data attributes such as functional annotations, expression levels, scores, synonyms, descriptions etc; 2) applying network data association rules to build the network and perform the visual annotation of graph nodes and edges according to associated data values. We demonstrated the advantages of these new capabilities through three biological network visualization case studies: human disease association network, drug-target interaction network and protein-peptide mapping network.

**Conclusion:**

The architectural design of ProteoLens makes it suitable for bioinformatics expert data analysts who are experienced with relational database management to perform large-scale integrated network visual explorations. ProteoLens is a promising visual analytic platform that will facilitate knowledge discoveries in future network and systems biology studies.

## Background

The concept of networks is ubiquitous in systems biology. In the past decade, high-throughput experimental techniques such as yeast 2-hybrid systems and mass spectrometry-based proteomics led to an influx of biomolecular interaction data in curated databases such as HPRD [1], DIP [2], and BIND [3]. Computational methods to predict protein interactions with domain interaction profiles [4], co-expression patterns [5], and term co-occurrences based on text mining [6] have also led to the development of databases such as OPHID [7], InterNetDB [8], UniHi [9], HAPPI [10], and STRING [11]. These databases support the transformation of biological network studies into essential biological data analysis tasks that include inferring global protein functions [12], assembling protein modules [13], integrating different Omics data sets [14], reconstructing biological pathways [15], predicting disease-relevant genes/proteins [16] and developing panel biomarkers [17].

Many network visualization software tools have been developed recently to help biological researchers visually query, annotate and analyze biomolecular network data. For example, Cytoscape [18] is one of the most commonly used software platforms that contains all basic functions for visualizing and annotating a network graph derived from protein-protein or protein-DNA interaction data. It has a robust graph layout engine that allows for automatic layout and manual control of network graph nodes and edges attributes corresponding to user annotation data. Cytoscape adopts an open and flexible software architecture that supports software plug-ins, which extends the core functionality of Cytoscape through third-party software extensions. VisANT [19] competes with Cytoscape by offering several built-in statistical functions to help users calculate several key network topological parameters and perform global real-time network analysis. WebInterViewer [20] uses a ultra-fast graph-layout algorithm that can scale up for manipulating the layout of a biomolecular interaction network up to tens of thousands of nodes on a desktop computer, while providing several network abstraction and comparison operators. The most recent feature-rich network data analysis software tool, Biological Networks [21], enables advanced bioinformatics users to integrate microarray data analysis with biomolecular interaction network analysis over a diverse set of database choices through powerful template-based query interfaces. Pathway Studio [22], which is available commercially, also uses powerful visualization engine and query interfaces, and allows its users to manage and access data stored in relational databases and to integrate biomolecular interaction data from its PubMed literature mining engine with other sources. In summary, current development trend is to equip users with extended ability to query and interpret existing experimental data, particularly those from "Omics" platforms, in the emerging context of biomolecular interaction networks.

Recent research in network biology has expanded beyond the study of protein-protein interactions or protein-DNA interactions, therefore presenting new challenges and opportunities for biological network visualization and analysis software. These networks are more complex, with heterogeneous types of biological entities spanning broad range of scales from molecular (i.e., DNA, proteins, metabolites), to super-molecular (i.e., gene ontology categories, protein complexes, pathways), to intercellular (i.e., signaling between different cell types), to tissue and physiological (i.e., individual disorder types) levels. For example, Goh *et al*. explored all known associations of disease phenotypes by representing disease phenotypes instead of molecular entities as nodes in a network graph [23]. They described two new types of biological networks, "disease interaction network" and "disease-gene network", in which the former represented disease names as nodes and disease associations at the molecular level (sharing > 1 disease genes between associated diseases) as edges, while the latter represented genes as nodes and gene associations shared in a common disease (shared > 1 diseases between associated genes) as edges. To characterize the global relationships between protein targets and all chemical drug compounds available today, Yildirim *et al*. [24] built a drug-target association network representing all known drugs and their targets recorded in the DrugBank database [25]. The network offered an intriguing view with "hot" drug intervention points (popular drug targets) and multi-targeted drugs clearly displayed. Analyzing the data in multi-scale biological networks is inherently more challenging than that of biomolecular interaction networks, primarily because the heterogeneous interacting biological entities may differ significantly in size, quality, complexity and annotation requirements, making it combinatorial more difficult to develop user interfaces that preserve usability and robustness at the same time. Few existing tools today can empower users to perform "visual analytics"–to discover novel information through visualization–for multi-scale biological networks.

To support multi-scale biological network visual analytics studies, new software tools must meet three basic requirements. First, the bulk of data should be managed by robust backend engines that support rich schemas such as relational database management systems (e.g., PostgreSQL, Oracle) or XML/RDF data stores (e. g, Jena, Piazza). Flat files quickly become unsustainable beyond one or two spreadsheets of custom user input data, due to lack of a standard schema and difficulty in combining information from separate spreadsheets. Second, iterative, exploratory and bi-directional data analysis capabilities to save temporary results and build visualization sessions on top of one another should be a pre-requisite. Many current software tools support only one-way information flow from data sheets to visualization, and therefore should be referred to as "visual annotation" or "visual display" tools instead of "visual analytic" tools. Third, visual querying languages, even if borrowed directly from SQL in relational database querying or SPARQL in semantic web based data querying, will become quite beneficial to advanced users, who have to filter different facets of biological networks and manipulate complex network analysis tasks, by automating tasks that are "menu-driven" or "mouse-click intensive". As Suderman *et al *recently surveyed, none of the 35 commonly used biological network visualization tools supported such query languages embedded directly [26].

We developed ProteoLens as a new visual analytic software platform for creating, annotating and analyzing multi-scale biological networks. When compared with existing biological network visualization tools, ProteoLens introduced a new set of design choices, which made it easy for bioinformatics expert data analysts work on large sets of biological networks and Omics data. There are three primary characteristics that distinguish it from existing network visualization tools. First, it supports direct database connectivity to Oracle and PostgreSQL database and SQL statements including both Data Definition Languages (DDL) and Data Manipulation Languages (DML). Users of ProteoLens can use the tool to iteratively prepare data stored in relational databases without leaving the visual analytic environment. Data from different tables in a complex relational database schema can also be queried on the fly to create networks at the appropriate level for exploration. Second, ProteoLens supports graph/network represented data expressed in standard Graph Modeling Language (GML) formats. Therefore, visual layouts performed in comparable software tools can interoperate with ProteoLens as long as they also support GML standards. This allows users to perform visual network analysis for data from heterogeneous sources that are syntactically represented in non-relational format. Third, it supports the decoupling of complex user interfaces for network visualization into two separate functional layers: data annotation and data visualization. The concepts of "node association rules" and "edge association rules" provide users with significant flexibility in choosing what data attributes (e.g., score, rank, description) to map to nodes or edges, and association visualization display options allow to select visual effects to represent values of these attributes later.

In the next several sections, we first describe ProteoLens implementation and then demonstrate how it can be used to enable multi-scale biological network-based research through three case studies.

## Implementation

**Figure 1:**
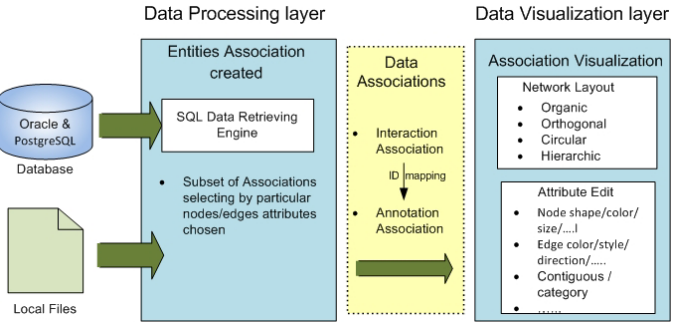
**An overview of the ProteoLens core architecture**. The design of ProteoLens decoupled the data processing and visualization presenting in two layers and the two layers communicated by the abstract Data Associations. The major components of ProteoLens are SQL data retrieving engine, network layout engine and graph attributes editing engine.

ProteoLens is a standalone software tool written in Java programming language. Its software architecture consists of two separate functional layers – a data processing layer at the backend and a data visualization layer at the frontend–connected by a network data association engine (Figure 1). Different from conventional network visualization software, where data preparation prior to visualizations is usually not supported by the software itself, the ProteoLens data processing layer allows network data to be pre-processed and integrated using built-in database management utilities. The data visualization layer at the frontend enables users to iteratively build and layout query-based (sub)networks and overlay them with visually displayed annotations as additional data sets are brought in. The network data association engine bridges the two functional layers by creating network data association rules (to be described next) between pre-processed data elements and identifiers of network nodes or edges. This design enables users to navigate between data management and data visualization iteratively until useful insights from the proper visualization are established.

### Network data association rules: the concept

Network data association rules represent a basic concept in ProteoLens design. A network data association rule defines a relationship between a network data attribute such as an edge or a node and other non-network data attributes such as a computed score or an expression value. Such an association rule establishes the mapping between data in the data processing layer and data in the data visualization layer. There are two types of association rules:

1) *Graph Node Association Rules*. For example, Rule *X*: {Protein ID} → {Protein Name}defines the network data attribute "Protein ID" as an identifying attribute for a network node further annotated with a node attribute "Protein Name". Note that the network attribute "Protein ID" and associated non-network attribute "Protein Name" may not necessarily be stored in the same database table and may be defined using a complex SQL query for visual data mapping purposes only.

2) *Graph Edge Association Rules*. For example, Rule *X*: {Protein ID A, Protein ID B} → {} defines the combination of two network data attributes, "Protein ID A" and "Protein ID B", as identifying attributes for a network edge without further annotations; whereas Rule *Y*: {Protein ID A, Protein ID B} → {Interaction Score} defines the combination of two network data attributes, "Protein ID A" and "Protein ID B", as identifying attributes for a network edge further annotated with an edge attribute "Interaction Score". Similar to graph node association rules, the network attributes and non-network attributes may come from different physical data structures.

### Data processing layer

The data processing layer is the place where biological data from different sources, including flat files, XML data and tabular data in relational databases, can be managed and converted from one format into another for subsequent analysis. In ProteoLens, users could specify the sources of data, pre-process data and make certain subsets of data available to the subsequent data analysis. Unlike conventional visualization software tools, ProteoLens supports full Structured Query Language (SQL)–including both Data Definition Languages (DDL) and Data Manipulation languages (DML)–for these tasks. The combination of DDL and DML is particularly powerful for network biological studies, since many network data association rules may require selected data sets (via DML) and nested definition of complex data structures (via DDL) by pulling data from many physical table locations. In ProteoLens, the data processing layer is implemented with the combination of GML data handler and Oracle 11 g/PostgreSQL relational database engines.

### Data visualization layer

The data visualization layer is the place where specified network data attributes and data association rules are converted to network layouts and network visual properties. The data visualization layer accepts network data association rules, lays out the drawing of networks as graphs, and visualizes network nodes and edges using graphical attributes defined in the network data association rules. In ProteoLens, the data visualization layer is implemented with a fully functional graph editor, which supports laying out the nodes and edges in the network and editing their graphical attributes such as colors and shapes with rules defined in network data association rules. ProteoLens supports multiple independent network views. In a network view, each associated attribute specified by the association rule can be added either as a node attribute or an edge attribute, depending on the association rule type. Any numbers of associations can be selected as annotation sources to modify the appearance of network nodes and edges. In ProteoLens, the graph layout is extended from yWorks Java package 3.0, a commercially available graph layout library.

## Results and discussion

### New features

**Figure 2:**
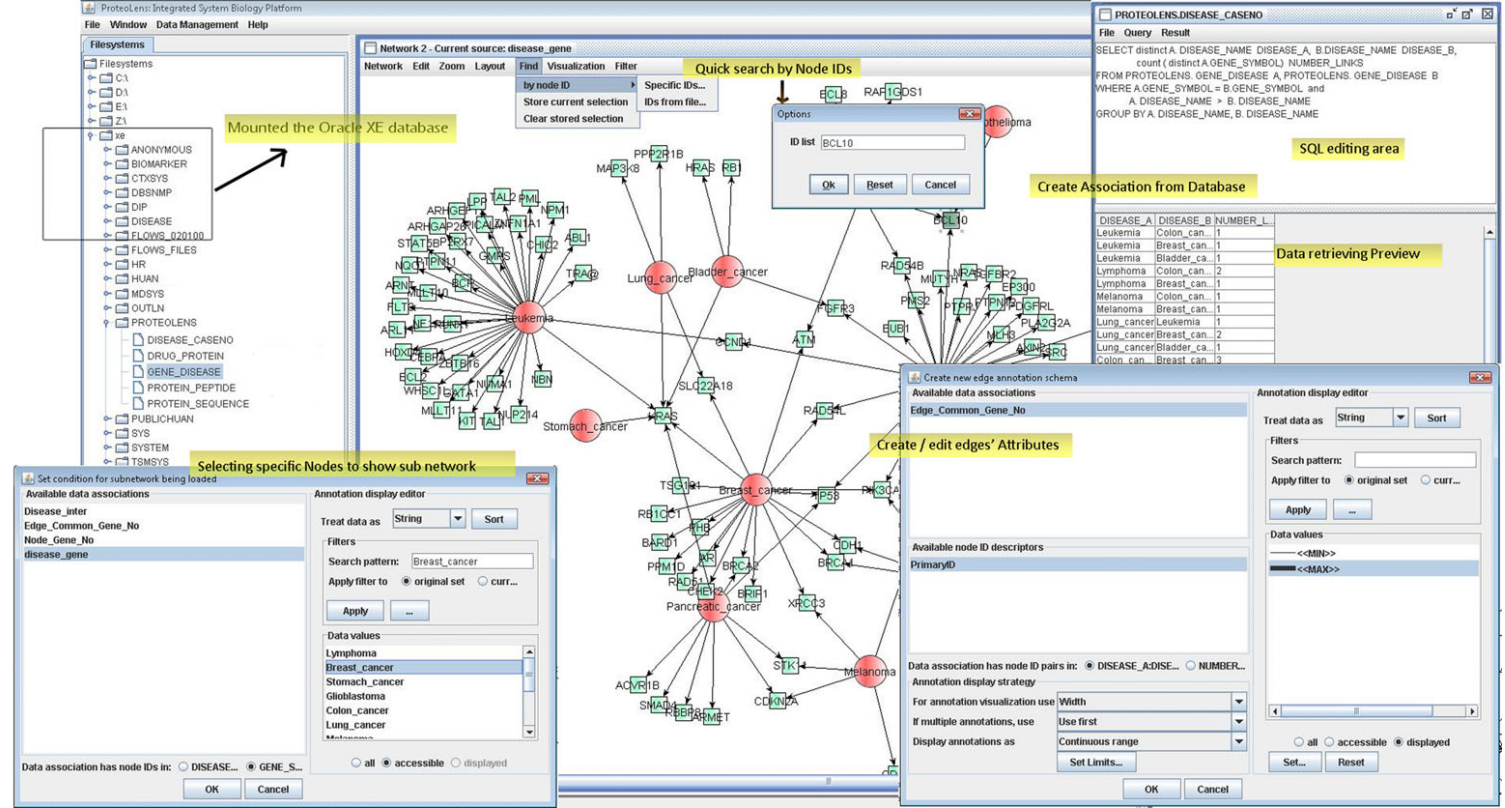
**The overview core functionalities of ProteoLens**. Some of the core functionalities of ProteoLens labelled in this figure: a) ProteoLens can access both the relational database and local file system, b) the SQL statement can be edited and run in the software environment for data association building, c) SQL-like for building the sub network by retrieving particular characters of nodes/edges, d) convenient quick query of the nodes in the network view and sub-network retrieving, and e) flexible and comprehensive annotation adding.

Figure 2 illustrates the core functionalities of ProteoLens. We compared ProteoLens with several existing popular visualization software tools, including Cytoscape, VisANT and BiologicalNetworks, and summarized the comparison in Table 1. We describe the features of ProteoLens in detail in this section.

**Table 1:** Compare ProteoLens against Cytoscape, VisANT and BiologicalNetworks.

	**ProteoLens**	**Cytoscape**	**VisANT**	**BiologicalNetworks**
**Graph manipulation**	yFiles	yFiles and GINY	In house	Cytoscape
**Laying out Network Algorithm**	force-directed, Radial Layout hierarchical, circular, orthogonal	More than 13 kinds of layout styles.	force-directed	grid, circular, force-directed
**Drawing appearance**	node shape/colour/border/label, edge colour/style/direction/label	node shape/colour/border/label, edge colour/style/direction/label	node shape/colour/size	node shape/colour/border/label, edge colour/style/direction/label
**Filters**	select nodes/links according to properties or using SQL statement for table attributes selecting directly	select nodes/links according to properties (SQL-like)	Several 'select' filters available	select nodes/links according to properties (SQL-like)
**Expand/Collapse nodes**	expand node neighbours	Plug-in	expand node neighbours	
**Database Incline**	Common Relational database	Plug-in	Predictome	PathSys
**System Requirements**	Java stand-alone	Java applet or stand-alone	Java applet	JSP (Java Server Pages)
**Save**	GML,XML session	GML,SIF	network with layout	save all work as projects
**Imports**	Text, GML, XML, Oracle or PostgreSQL	text, GML, expression matrix, OBO	PSI-MI, BioPAX, KGML, network relations (text)	microarray data (Stanford,Affymetrix,TIGR,GenePix), SBML, SIF, PSI-MI, BioPAX
**Exports**	JPEG,BMP, GML network relations, Node lists, selections node lists (text)	graphical file, SVG, GML, network relations (text)	PSI-MI, BioPAX, SVG, JPEG, network relations (text)	GIF, JPEG, SWF, PDF, PNG, PostScript, RAW, SVG, BMP
**Comments & other features**	Embedding the SQL query make its software more flexible to suit powerful bioinformatics experts usage	The importance of Cytoscape is its solid support for plug-in, growing number of which is available.	Statistics ability for topological characteristic analysis and integrating several biological database	Integrated visualization and analysis of expression data.

*A summary of attributes of Cytoscape, VisANT and BiologicalNetworks as presented in detail by Matthew Suderman *et al *in review. [26]

#### a. Relational and XML data support

Currently, ProteoLens supports two types of physical data sources: tab-delimited text files on the local file system and tables/views in relational tables managed by Oracle 10 g or PostgreSQL 8.x database management systems. A user can manage and query the data stored in the relational database, create network data association rules from the view, and immediately make the rule available for visual annotation. Since only meta-data are stored when a data association is created, the query execution can be performed in real time of visualization against the underlying complex data structure in the database. This design allows for an efficient data retrieval and analysis, and saving of the data file and workspace resources. Almost infinite configurations of data views can be created from multiple underlying data sources, and be used for building complex integrated visualizations. ProteoLens also supports semi-structured data format in Graph Modeling Language (GML) – the standard file format in the Graphlet graph editor system, for non-relational graphs. Network visualization is created in a view can be saved in a GML file, thus allowing for reopening and further editing in a new session, or data exchange without relational databases. The network view can be exported as a JPEG or PNG file. The user can import and manipulate any network data using standard GML file formats in addition to structured data stored in the relational databases. ProteoLens stores every data association in a session configuration XML file. Users can save the session and recommence their analysis at any time.

#### b. SQL-based visual data analysis

ProteoLens supports direct database connectivity through Java Database Connectivity (JDBC) to Oracle and PostgreSQL database tables and views, and the entire set of database operations can be specified using full SQL statements including Data Definition Languages (DDL) and Data Manipulation languages (DML). This extends the range of data that expert users may bring into later network visualizations for annotation and visual exploration tasks. Users of ProteoLens can iteratively prepare data stored in relational databases without leaving the visual analytic environment. Data from different tables in a complex relational database schema can also be queried on the fly to create networks at the appropriate level for exploration. SQL queries are also used to present "views" of different underlying database tables for network data associations, therefore making it possible for users to perform all visualization pre-analysis without leaving the ProteoLens platform.

#### c. Flexible network data visual annotation

In ProteoLens, users can explicitly define network data association rules. As described earlier, the IDs of the nodes in the network are used for attaching multiple attributes in the rule for subsequent visual annotations. The annotation of an edge is based on the mapping of attributes in the rule identified by two interacting node IDs for each edge. Graphical attributes currently available for automatic node annotations are: label, size, shape, and fill color. The latter annotation allows mapping multiple or multiple-valued properties (multiple colors per node object), in which case pie-chart style filling will be drawn. Graphical attributes currently available for automatic edge annotation are: line style, width, color, and text label. There are two types of mappings between attributes and visual properties: 1) the *categorical mapping *type that allows displaying attributes with discrete set of specific property values (for instance, using protein ID → GO molecular function association rule, and requesting all nodes annotated as a "kinase" or a "phosphatase" to be colored in red); and 2) the *continuous-range mapping *type that allows displaying properties with continuous numerical values (*e.g*. expression levels, or interaction confidence scores), using color gradients, shape sizes or line widths.

The use of declarative SQL to specify how data should be managed, pre-processed, associated, and then subsequently mapped to visual properties is characteristic of ProteoLens. A user can use SQL queries to specify and store "associations" between nodes/edges and other attributes. These associations can be used to visually annotate large displayed networks using node/edge shape, size, weight, color and text. This gives users more choices and flexibility than any custom-built annotation user interface for complex visual network analysis.

#### d. Sub-network manipulations

Users of ProteoLens can conveniently specify sub-networks based on existing networks to conduct studies in a specific biological context. Users could specify what types of nodes or edges to include in the sub-network according to set of qualifying conditions. For example, a user may retrieve all the interactions where at least one of the partners is annotated as "cell-cycle related" proteins. This does not require bringing into the view a huge biomolecular network in its entirety and then filtering/zooming in onto its part. Neither this specification requires users to prepare the data outside the ProteoLens visualization software platform; instead, users could write a SQL statement to first create a relational view between protein nodes and gene ontology annotations and then to create a new network data association of "nodes", with which a new sub-network could be retrieved from the original network and annotated. This approach can be imagined as a huge underlying biomolecular network stored in the backend databases and/or files and integrated (logically) by the virtue of data associations. Only the relevant part of the network is physically pulled into the visualization layer for detailed examination. Such visualized sub-networks are not "final" either but could be gradually updated by iteratively using additional queries to bring more data from different sources into the view, until the final complex network is built. In comparison, Cytoscape does not support a network filtering feature and requires the entire network for visual data analysis to be pre-formatted properly and imported from the input data files. A Cytoscape software plug-in, Bubble Router, has became available recently; however, it allows only creating a sub-network with one-pass filters, which cannot be iteratively extended to fit the exploratory nature of visual analytic operations.

### Case studies

To demonstrate the new functionalities of ProteoLens, we show several case studies that demonstrate how the software is used to solve real-world biological problems.

#### Case study 1: human cancer association network

Decade-long study of disease-causing genes has generated a comprehensive set of "disease disorders – genes" relationship pairs (also referred to as the "diseasome"), which are represented in the OMIM morbidity map [27]. Goh *et al*. recently showed a global view of the "human disease network" (HDN), which included 22 disease disorder classes, 1284 disease disorders, and 1777 disease genes [23]. In the HDN, nodes represented disease disorders, while edges represented the presence of at least one common gene between two connected disease disorders. The study of HDN showed how diseases related to one another and formed major disease clusters connected by underlying shared molecular entities. The initial construction of the network for HDN, however, was labor-intensive, since preparation of the disease-gene association file and other gene/disease annotation files needed to be processed separately with different software tools before visualizations were to be performed.

In this case study, we show how to reproduce a similar human disease network with ProteoLens, using data for 13 common types of cancers derived from the OMIM database, papers by Goh *et al*, and public biological databases [23,27]. The visual analytic process can be divided into four steps, network data pre-processing, network data association rule specifications, initial network visualization, and iterative visual data analysis, all without leaving the software platform:

##### Network data pre-processing

**Figure 3:**
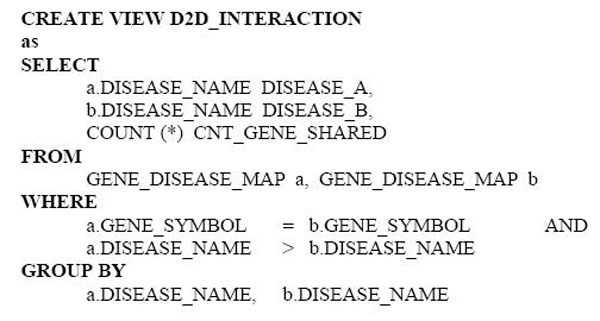
**SQL statement for identifying disease-disease association**. The SQL statement created a view recording the relationship of two individual diseases as "associated" if they shared at least one common disorder gene.

At the beginning of the analysis, only one database table–the GENE_DISEASE_MAP table–is available. The table contains the pairing relationships between disease disorders and genes. From this table, we could define the relationship of two individual diseases as "associated" if and only they shared at least one common gene implicated in both diseases. The SQL to create such a specification is quite straightforward: (Figure 3)

**Figure 4:**
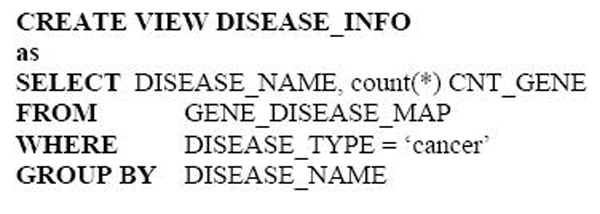
SQL statement for counting the genes involving in every disease.

The constructed view put together three attributes. The first and second attributes represent paired cancer diseases that share at least one common disorder gene. The third attribute represent the total count of shared genes between the shared diseases. To create an annotation for cancers and total count of genes implicated in each cancer, we can write the following SQL statements inside ProteoLens: (Figure 4)

##### Network data association rule specifications

Here, we assign visual attributes of interest to either network nodes or edges, using network data association rules. In this case study, we can create the following sample association rules using ProteoLens:

• *Disease_gene_implicated*: {Disease_Name} → {Cnt_Gene}

• *D2D_gene_shared*: {Disease_A, Disease_B} → {Cnt_Gene_Shared}

Note that network data association rules must involve mapping from either the node (identified by Disease_Name) or edge (identified by Disease_A, Disease_B) to an annotation attribute such as "Cnt_Gene" or "Cnt_Gene_Shared". The former data association rule is "node-styled" and the latter data association rule is "edge-styled", since they provide annotations (attributes) for nodes and edges, respectively.

##### Initial network visualization

The final construction of the human cancer association network in ProteoLens is now simplified. First, we lay out the basic network disease pairing information from "D2D_INTERACTION"; then, we apply all the node-style network data association rules to the annotation of "nodes" and all the edge-style network data association rules to the annotation of "edges". In this case study, we chose to represent the total numbers of genes implicated in a given disease as sizes of the disease nodes, and the total numbers of genes shared between two diseases as the edge widths.

##### Iterative visual data analysis

**Figure 5:**
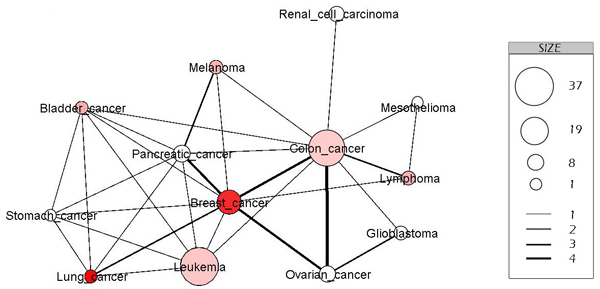
**Disease-disease association network**. This is a sub network of the cancer disease association network, built by retrieving 13 kinds of popular cancer. In this representation, the node is a kind of cancer, and if two kinds of cancer have common genetic disorder genes, there is an edge connecting them. The size of nodes indicates the number of cancerogenic disorder genes and the color of nodes indicates the number of cases in 2007 in the U.S; dark red indicates more cases, light red indicates less number, and white indicates less statistic data. The width of edge indicates the number of common genetic disorder genes of two kinds of cancer disease.

ProteoLens supports iterative visual analysis by allowing additional visual information to be captured as network nodes/edges annotations later. In this case study, after examining that breast cancer is well studied (with many mapped genes) and lung cancer is not, we decided to further incorporate first-time incident frequency during 2007 in the U.S., by retrieving relevant statistics from the American Cancer Society [28] and annotating disease names represented as network nodes with color gradients from white to red. The final view of the annotated network is shown in Figure 5. The figure reveals intriguing insights into the relations of the human cancers to each other. Interestingly, lung cancer studies are found to be under-represented for all cancers, given its large number of new incident rates; the discovery opportunities for lung cancer seem huge.

#### Case study 2: compound-target interaction network

Different from conventional network biology studies or case study #1, this case study is concerned with two different types of biological entities–chemical compounds and drug target proteins. We attempt to understand the specificity of drug compound actions and to visualize potential drug targets for major diseases.

Using ProteoLens, we created drug-target network visualization, using hierarchical layout and two different node shapes to represent drugs and protein targets separately. In Figure 4A, we show a snapshot of the network, which contains all the drug compounds developed for ACM2_HUMAN and its direct interacting protein partners. ACM2_HUMAN is an acetylcholine binding receptor and a member of the G-protein-coupled receptors (GPCR) protein family–a major class of current drug targets that accounts for more than 50% of known contemporary drug compounds [29].

**Figure 6:**
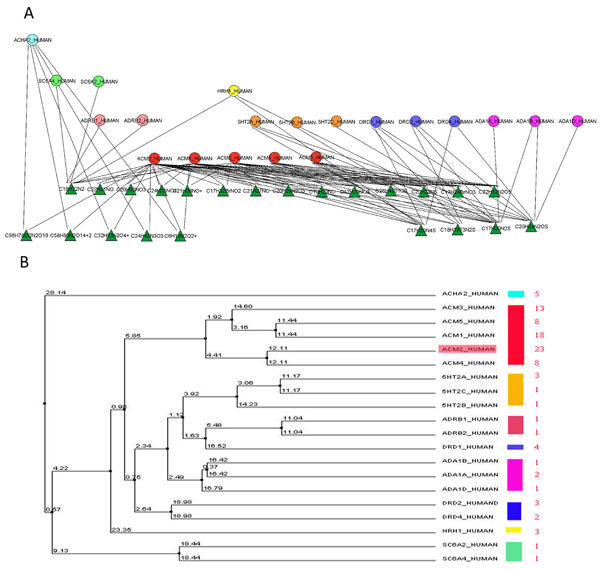
**Compound- protein target interaction network**. a) The compound-protein target interaction network is drawn by the ProteoLens hierarchical layout. b) The evolutionary tree of all the target proteins is drawn by ClustalW2 . For every protein sub family marked with a color, the node color in A corresponds to the color bar in B.

The entire protein-drug interaction data was downloaded from DrugBank [25] and stored in Oracle 10 g database tables. It is interesting to note that target proteins could be visually clustered, and the clustering relationships correlate well with the evolutionary relationship defined by separate alignments of protein sequences (see Figure 4B). While a few ACM2_HUMAN interacting proteins such as SC6A1_HUMAN, SC6A2_HUMAN and ACHA2_HUMAN are not members of GPCRs, all other proteins belonged to GPCRs. ACM1_HUMAN to ACM5_HUMAN are acetylcholine receptors. As shown in Figure 6, the results suggest that proteins with similar phylogenetic profiles tend to share similar core set of drug compounds, perhaps due to similarities of underlying protein structures in the proximity of the functional site. The visualization of such drug-target network opens up new "network pharmacology" study opportunities, in which a drug may be evaluated for its ability to find multiple "targets" related to a specific biological sub-network [30]; while the effects of drug compounds may also be evaluated in the context of common structures of all interacting target proteins. This type of visual network studies could help users develop novel perspectives for drug designs and/or protein target validations.

#### Case study 3: peptide-protein mapping networks

In this case study, we apply ProteoLens to the study of mass spectrometry (MS) based proteomics. In each tandem mass-spectrometry experiment, many partially Trypsin-digested peptides can be detected by the MS/MS spectrometers and identified by MS analysis software. The software normally aims to identify all the peptides from MS spectra and to map them unambiguously to proteins in the sample. Traditional MS analysis software identifies proteins from peptides by directly mapping them to the most common protein isoforms found in the pre-computed MS search database; therefore, incomplete results may arise, especially in cases where common peptides may be shared by two or more protein isoforms.

**Figure 7:**
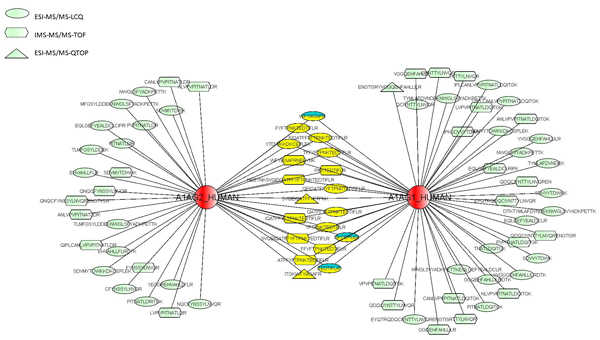
**MS proteomic peptide-protein mapping network**. The blue color marking nodes are the original common peptides of the two proteins, and the yellow ones are newly discovered common peptides. The peptides' nodes are marked in three kinds of shape indicating different MS experimental platforms.

**Figure 8:**
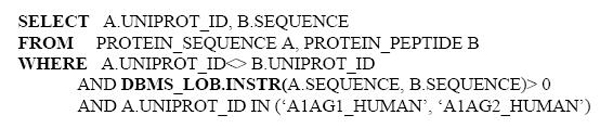
SQL statement for identifying the protein-peptide relationship.

In Figure 7, we show how ProteoLens could be used to help establish all the relationships between found peptides and possible protein isoforms that they may link to, in the HIP2 database–an online database that collects all experimentally identified proteins and peptide-mapping evidence in normal human plasma[31]. Two proteins, A1AG1_HUMAN and A1AG2_HUMAN, are shown in the visualization network. By writing SQL inside ProteoLens, we identify all the potential protein-peptide relationships: (Figure 8).

In the peptide-protein mapping network shown, the common peptides identified and mapped to either protein in the original experiments are colored green, whereas newly mapped peptides are colored yellow. We also used different node shapes to annotate peptides identified from different MS instrument types, e.g., IMS-MS instruments, LC-MS/MS instruments or MALDI-MS instruments. By visualizing the raw data in the protein-peptide network, we can see that both protein isoforms are found in human plasma, since both common peptides and protein-specific peptides are found and mapped. Interestingly, IMS-MS instruments are seen to resolve only one protein isoform "A1AG1_HUMAN", suggesting that either the platform could be biased towards identifying certain types of peptides or the search database used for this experiment might have not contained A1AG2_HUMAN. ProteoLens makes it easy for users to explore different hypothesis and continue scientific explorations through iterative network visual data analysis.

## Conclusion

We developed ProteoLens as a multi-scale network visual analytic software tool for advanced network biology studies. It is built on robust software architecture that supports flexible network data association specification using rules, integrates data processing through relational databases and GML data files, and scalable data visualization through layered annotations. It is intended for advanced bioinformatics users who manage large existing sets of biological data in the Oracle or PostgreSQL databases, and who are skilled in SQL programming. ProteoLens is by far the first bio-molecular network visualization software with full SQL support. ProteoLens enables iterative visual layout, annotation and exploration of bio-molecular networks. It effectively liberates advanced data analysts from the burden of data preparation and processing prior to generating visualization, and thus helps to better concentrate on the scientific visualization itself. The support for both "network browsing" and "network querying" operations makes ProteoLens a promising visual data analytic and visual data mining tool for hypothesis-driven network biology studies. With future releases of ProteoLens, we plan to add open Application Program Interfaces (APIs) so that 1) Proteolens can interoperate with other software tools in bioinformatics, and 2) third-party plug-ins could be developed to accommodate expanding user community needs.

## Availability and requirements

**Project name: **ProteoLens

• **Project home page: **

• **Operating system(s): **The software is platform independent and can run anywhere Java Virtual Machine runtime is available. An installer is provided for Windows NT/XP/2003/Vista users.

• **Programming language: **Java

• **Other requirements: **Java Runtime Environment (JRE) version 1.5 or above is required.

• **License: **free software license to all users.

• **Any restrictions to use by non-academics: **Non-academic users can freely use the software for research purposes. Non-academic users cannot redistribute, modify, reverse-engineer, or resell the software for commercial purposes.

## List of abbreviations used

**GINY**- Graph Interface library; **SQL **-Structural query language; **PSI-MI **– Proteomics Standards Initiative – Molecular Interactions.

## Competing interests

The authors declare that they have no competing interests.

## Authors' contributions

JYC is the principal architect and custodian of the software. JYC and AS conceptualized the multi-scale biological network visualization ideas together, designed computing architecture, and implemented the software in 2003–2006. TH and SHH took over the software improvement and maintenance tasks since then by rewriting significant portions of the software, developing new documentations, testing it on different platforms, developing a set of case studies. TH outlined the paper and all authors have read and approved the final version of the manuscript.
